# 

**Published:** 1918-09-28

**Authors:** 


RATES OF SUBSCRIPTION :

Twelve months, 10/10; Six months, 5/5; Three months, 2/9, post free to any address in the United Kingdom. Abroad, Twelve months, 13/-; Six months, 6/6; Three months, 3/3, post free. **THE HOSPITAL "Index" to Volume LXI., October 1917 to March 1918, is now ready, price 3½d. per copy, post free.** Address The Manager, THE HOSPITAL, "The Hospital" Building, 28/29 Southampton Street, Strand, London, W.C. 2.

### Queen Alexandra and the London Hospital.

To prepare for Queen Alexandra's seventy-fourth birthday, which takes place on December 1, a fund on behalf of the London Hospital is being collected. Nor are the admirers of Queen Alexandra and of the London Hospital confined to people in this country. A similar effort for a similar purpose, according to the Copenhagen correspondent of the *Daily Telegraph*, is being made in Denmark, under the presidency of Dr. Reyn, the chief of the Finsen Light Institute, and a number of prominent people in every walk of life are supporting Dr. Reyn. There is reason to think that the fund will be worthy of the occasion, a fit compliment to Her Majesty, and therefore a real help to the London Hospital.

